# Is Isocyanate Exposure and Occupational Asthma Still a Major Occupational Health Concern? Systematic Literature Review

**DOI:** 10.3390/ijerph182413181

**Published:** 2021-12-14

**Authors:** Elie Coureau, Luc Fontana, Céline Lamouroux, Carole Pélissier, Barbara Charbotel

**Affiliations:** 1 UMRESTTE, UMR T 9405, Université Lyon 1, Université Gustave Eiffel—IFSTTAR, Domaine Rockefeller, 8 Avenue Rockefeller, 69008 Lyon, France; coureau.elie@hotmail.fr (E.C.); celine.lamouroux@chu-lyon.fr (C.L.); 2 CRPPE-Lyon, Centre Régional de Pathologies Professionnelles et Environnementales de Lyon, Centre Hospitalier Lyon Sud, Hospices Civils de Lyon, 69495 Pierre Bénite, France; 3 Hospital University Center of Saint-Etienne, Université Lyon 1, Université de St Etienne, IFSTTAR, UMRESTTE, UMR T 9405, 42005 Saint-Etienne, France; luc.fontana@chu-st-etienne.fr (L.F.); carole.pelissier@chu-st-etienne.fr (C.P.); 4 Service de Santé au Travail, Centre Hospitalier Universitaire de Saint-Etienne, 42005 Saint-Etienne, France

**Keywords:** occupational asthma, isocyanates, systematic review

## Abstract

Isocyanate, whose disease-inducing mechanism is poorly understood, with poor prognosis, is widely used. Asthma is the most frequent manifestation of prolonged exposure. We assessed the evolution of the incidence of isocyanate-induced occupational asthma over time. PubMed and Cochrane databases were systematically searched for studies published since 1990 that assessed the relationship between occupational exposure to isocyanates and asthma. We identified 39 studies: five retrospective cohort studies, seven prospective cohort studies, three of which were inception cohorts), seven observational cross-sectional studies, five literature reviews, two case series, and 13 registry studies. The incidence of occupational asthma secondary to isocyanate exposure has decreased from more than 5% in the early 1990s to 0.9% in 2017 in the United States. Despite the wide use of optimal collective and individual protection measures, the risk of occupational asthma has stabilized. Occupational asthma risk can be assessed with good sensitivity using self-questionnaires and pulmonary function tests. Occupational avoidance should be implemented as soon as possible after the first symptoms appear because the prognosis becomes increasingly poor with the persistence of exposure. It is now necessary to study specifically cutaneous sensitization to isocyanates and to define what protective equipment is effective against this mode of exposure.

## 1. Introduction

Organic isocyanates are highly unstable chemical compounds, with at least one isocyanate function (-N=C=O), due to this function which is highly unsaturated. This explains their widespread industrial use, the complexity of the mechanisms of action of the induced pathologies as well as their poor prognosis, both on the medico-occupational and medico-social levels [1]. Polyurethane, in the family of plastics, is formed when they react with polyol. They are used in many sectors of activity, such as the automotive industry, footwear, construction, foundries, tanneries, the electronics industry, the production of molded parts, printing, painting, maintenance [2].

The most used isocyanates in industry are: TDI (2,4 and 2,6 toluene diisocyanate, the most harmful because of its high volatility at room temperature), HDI (hexamethylene 1,6-diisocyanate, also volatile at room temperature), and MDI (diphenylmethane 2,4-diisocyanate) (1). Others such as NDI (naphthalene diisocyanate) and IPDI (isophorone diisocyanate) are also used in industrial settings.

The main route of contamination is respiratory, as these products are mainly used by spraying, which leads to the presence of isocyanates in the air in the form of aerosols.

Asthma, the most common disease caused by prolonged exposure to isocyanates, is one of the most common occupational respiratory diseases in industrialized countries [3]. Asthma is a respiratory disease characterized by wheezing and coughing and chronic inflammation of the airways that can cause exacerbations and progressive lung function decline. The diagnosis is confirmed by an alteration in peak expiratory flow on spirometry. In France and other industrialized countries, in 2016, the average annual incidence of occupational asthma was estimated at 30.3 cases per million workers, i.e., an average annual decrease of 15.8% [3]. However, this incidence is known to be underestimated and should be considered as the minimal rate [4].

Although flour is the most frequent etiological agent for occupational asthma, isocyanates are the main chemical cause in France and are responsible for between 8 and 17% of cases [3,5]. Elsewhere the prevalence varies between 5 and 10% of cases, depending on the country [1].

The aim of this systematic literature review was to assess the evolution of the frequency of occupational asthma due to isocyanate exposure over time. 

## 2. Materials and Methods

### 2.1. Information Sources and Search Strategies

PubMed and the Cochrane Library were searched from 1990 to 15 April 2021. The following reference keywords were identified using the terms ‘occupational asthma’ and ‘isocyanates’ in the Medical Subject Headings Terms (MeSH Terms) tool: asthma, occupational; work-related asthma; WRA; isocyanates.

The following search strategies were used:

- PubMed
  ○ ((isocyanate * [MeSH Terms]) OR (isocyanate * [Title/Abstract])) AND ((((asthma, occupational [MeSH Terms]) OR (asthma, occupational [Title/Abstract]) OR (work related asthma [Title/Abstract]))
- Cochrane
  ○ #1 MeSH descriptor: [asthma, occupational] explode all trees
  ○ #2 (asthma, occupational): ti, ab, kw
  ○ #3 MeSH descriptor: [Isocyanates] explode all trees
  ○ #4 (isocyanate *): ti, ab, kw
  ○ #5 #1 OR #2
  ○ #6 #3 OR #4
  ○ #7 #5 AND #6

### 2.2. Selection of Articles

Eligible publications were published between 1990 and 15 April 2021 in English or French and reported the diagnosis of *de novo* occupational asthma with work-related exposure to isocyanates. We excluded publications reporting results from animal studies, clinical trials, morbidity and mortality assessments, non-occupational asthma, studies of long-term evolution of occupational asthma or after occupational avoidance, studies of the effects of comorbidities on asthma severity, studies and recommendations on legal exposure values, studies of the correlation between dose and the severity of symptoms, studies and recommendations on personal protective equipment, comparative studies of diagnostic strategies, genetic studies, and analysis of biomarkers.

The records were initially screened on the titles and abstracts then the PDFs of selected publications were screened to identify those that corresponded to the inclusion criteria.

The search and analysis were carried out by EC with verification by the co-authors in compliance with PRISMA Guidelines [6].

### 2.3. Data Collection and Analysis

Data on the study design population characteristics, country, isocyanate studied, target sector, size of the study population, duration of follow-up, diagnostic criteria for asthma, the results, evidence of work-relatedness, and the main limitations of the study were extracted into tables and analyzed. 

## 3. Results

Of 650 records identified from PubMed and the Cochrane Library, 189 were selected from the initial screen. The second screen resulted in the selection of 39 articles for inclusion in our systematic review: 12 cohort studies (five retrospective and seven prospective cohort studies, of which three were inception cohorts); seven observational cross-sectional studies; five literature reviews; two case series; and 13 registry studies. 

The majority of studies were conducted in the United States, Commonwealth countries and Europe, demonstrating that it is an international issue in industrialized countries.

The main sectors of activity concerned were the automotive industry (seven studies), chemical industry (six studies), wood industry (two studies), foundries and molding plants (two studies), other industries (four studies).

### 3.1. Automotive Industry

We identified seven studies: four cross-sectional studies, two cohort studies and a case series published between 1996 and 2017 that reported the use of a variety of isocyanates in the automotive industry [7,8,9,10,11,12,13] (Table 1).

In 1996, Simpson et al. reported that 41% of workers had altered PFT after 10 weeks of exposure to BIC (butyl isocyanate) prepolymer in the absence of IPE. However, these disorders were resolved after the introduction of air-supplied masks [7].

Other studies reported a proportional relationship between the duration of exposure to isocyanates and the frequency and intensity of respiratory symptoms [8,9,10,11]. Small and confined workspaces were associated with an increased risk and the use of an air-supplied mask was associated with reduced risk [12]. Poor respiratory symptoms and the presence of specific IgG were reported to be prognostic markers for the development of occupational asthma [13]. The incidence of occupational asthma was estimated to be about 10% among car-body painters, and this increased with exposure to 23% in subjects who had been exposed for more than 20 years [8].

### 3.2. Chemical Industry

Six studies reported results for chemical industry employees: five cohort studies and one case series, covering the period from 1999 to 2017 (Table 2). TDI and HDI are the main isocyanates used in this industry [14,15,16,17,18,19].

A case series study by Kraw and Tarlo published in 1999 reported an incidence of occupational asthma of 2.5% over five years of regular exposure, without information on the use of IPE [14]. A cohort study, using data from employees’ medical files, found 6.4% (19/313) suspected cases of occupational asthma (without a formal diagnosis) in employees who had been exposed to TDI for at least five years [15]. A higher incidence of occupational asthma in recently recruited employees who had previously been exposed to isocyanates was also reported.

In two studies conducted in companies where air measurements did not show a reading above the exposure limits, no occupational asthma cases were identified after 20 years of retrospective follow-up [16,17]. This finding was confirmed by a shorter inception cohort study (18). The proportion of employees with respiratory symptoms suggestive of occupational asthma (without a formal diagnosis) was 15% among exposed individuals, even if the exposure limits were not exceeded. In the absence of biomonitoring, the possibility that the employees were exposed to occasional peaks of isocyanates, without thus causing asthma cannot be excluded [17].

The cohort study by Collins et al. [19] estimated the incidence of occupational asthma at 0.009 cases/person–year in a population of workers exposed to TDI in different US companies. However, this figure is probably underestimated due to the non-inclusion of contract workers and the absence of formal diagnostic criteria.

### 3.3. Wood Industry

We identified two cohort studies in the wood industry which used MDI [20,21] (Table 3). One study by Petsonk et al., published in 2000, reported no cases of occupational asthma, but 12% of the employees were symptomatic after two years of exposure to MDI [20]. The results from this study also suggested a link between the appearance of work-related breathing difficulties and the reported intensity of exposure, smoking status, poor use of respiratory IPE or exposure to skin splashes. The second study by Wang and Petsonk, published in 2004, aimed to identify early symptoms following daily exposure to MDI. The authors observed significant onset of wheezing (at rest), cough and chest tightness [21]. Daily productive cough was associated with smoking status, not occupational exposure. The appearance of new symptoms after two years of follow-up was reported for 13% of the exposed workers [21].

### 3.4. Foundries and Molding Plants

We identified two cohort studies in the foundry sector, which is also known for its use of isocyanates, particularly in the manufacture of sand molds [22,23] (Table 4). In 1993, Bernstein et al. published a study of employees exposed to MDI for three years and showed a prevalence of occupational asthma of about 1% (three cases in 243 employees) under standardized working conditions, with continuous monitoring that did not exceed the 0.05 ppb threshold, and work carried out in a closed environment with ventilation [22]. The prevalence was inversely proportional to the total exposure. The prevalence of mildly irritated respiratory symptoms was 11%, which is consistent with our current knowledge of occupational risk.

A retrospective cohort, published in 2002, reported a prevalence of chest tightness of 16% over five years, under similar exposure conditions, i.e., undetectable levels. There were no cases of occupational asthma [23].

### 3.5. Other Industries

Four other studies were identified in sectors of activity that traditionally use fewer isocyanates (textiles industry, bone glue factories and various industries) [24,25,26,27] (Table 5). These studies reported contradictory results. A retrospective cohort by Baur et al., published in 1994, analyzed data for 1780 employees in various industries and, with a rigorous diagnostic approach using an isocyanate-specific inhalation test, estimated that exposure to MDI or TDI had no effect on lung capacity in previously healthy subjects and a moderate impact in asthmatic subjects [25]. However, a cross-sectional study reported 13% of exposed subjects had positive specific inhalation test results and 26% had symptomatic disease [24].

Ten years later, Al-Batanony et al. reported a relative risk of four in subjects exposed to TDI with a concomitant decrease in FEV1 and FVC proportional to the duration of exposure [26]. Another study reported the prevalence of exposure among workers in Australia in 2014 [27]. The authors estimated that 3% of the Australian working population was exposed to isocyanates in the workplace. The three most exposed occupations were painters (34.7%), woodworkers (24.4%) and construction workers (17.7%).

### 3.6. Literature Reviews

We identified five literature reviews that assessed all types of isocyanates in a varied population of workers from different industries [28,29,30,31,32] (Table 6). In 2000, Van Kampen et al. estimated the prevalence of all respiratory diseases attributable to 5% isocyanates at 5% [28]. 

Two other reviews estimated that, despite a drastic increase in the production and use of isocyanates, there has been a clear decline in the incidence of occupational asthma over the past 50 years from more than 5% of exposed workers before 1980 to less than 1% since 2000 [29,30]. They attributed this decline to the widespread use of individual protective equipment in industry.

Another study by Jarvis et al. suggested that the occupational asthma hazard of isocyanate is proportional to the number of NCO groups that the molecule contains, but the authors were not able to exclude potential interaction between different substances [31].

A 2007 review confirmed the decrease in the incidence of isocyanate-related occupational asthma due to the sharp decrease in exposure by the respiratory route but warned of the potential role of skin exposure in the occurrence of respiratory signs [32].

### 3.7. Epidemiological Public Health Registers

Occupational asthma can be monitored in registry studies (Table 7). We identified 13 publications on national health monitoring registers in the United Kingdom, France, South Africa and Belgium published from 1992 to 2020 [33,34,35,36,37,38,39,40,41,42,43,44,45]. The data from these registers suggest an overall decrease in occupational asthma cases over time which is indexed on the industrialization rate of each region, as well as a decrease in the proportion of occupational asthmas related to isocyanates. The incidence of occupational asthma in the general working population was reported to range from 13 to 25 per million workers [35,37,40,42,43]. In highly industrialized regions such as the West Midlands in England, the incidence was more than 40 per million workers [33]. The incidence was much higher for jobs at risk of exposure, in particular car bodyworkers, with more than 1500 cases per million workers in the United Kingdom before 2000 [33,34], and 326 cases per million workers in France in 2002 [38].

Isocyanates were reported to be the leading cause of occupational asthma in the UK [33,36,39,41] and the second in France, after flour [38,44]. However, there has been an overall decrease in the percentage of occupational asthma attributable to isocyanates in all industrialized countries [34,45]. These observations were reported to be correlated with an increasingly systematic avoidance in the workplace after diagnosis [39].

## 4. Discussion

The objective of this literature review was to assess the risk of occupational asthma related to chronic exposure to isocyanates. Measuring the impact of various prevention and awareness strategies on the incidence of occupational asthma and its evolution over time remains an important issue in occupational medicine and health, as these compounds are still widely used in many industrial sectors.

Out of 650 records identified from PubMed and the Cochrane Library, 39 articles were selected following the PRISMA Guidelines, including studies from several industrial sectors, five literature reviews and 13 registry studies.

The incidence rate of isocyanate-induced occupational asthma in the US has fallen from 5% before 1980 to 0.9% in 2017 [19]. This decrease in risk has undergone two turning points: firstly, there was a clear and sharp decrease in the number of new cases reported at the start of the 2000s. The generalization of respiratory prevention measures in industry over the last 40 years is probably a major factor in this evolution [29]. In 1991 in France, 142 cases were compensated, corresponding to an incidence of one case per 100,000 employees. In 2016, the number of cases compensated fell to 23, i.e., an incidence divided by 6.5 [46]. Since 2007, the decrease in annual incidence appears to have slowed down considerably, with 31 cases recognized per year on average [46].

This observed decrease in incidence may be explained by the under-reporting of pathologies or by the delay in diagnoses and in declaring the case as an occupational disease. However, this decrease could also be explained by the reduced use of isocyanate-containing products or the more effective implementation of preventive measures described in labor laws in many countries. Nevertheless, the current preventive strategies, which are now widely used and almost optimal, seem to have reached their limits.

The results of this literature review are consistent with our current knowledge. We observed that the risk of occupational asthma is still present when isocyanates are handled, despite the use of appropriate individual and collective respiratory protection measures. We also noted that the main respiratory symptoms (resting dyspnea, coughing, chest tightness and wheezing) usually appear without delay and are mostly resolved when exposure is stopped [21], although in the studies analyzed here, proven cases of occupational asthma after several years of isocyanate exposure were not consistently reported, probably because of the short follow-up time in the studies. Therefore, we assessed the incidence of respiratory symptoms suggestive of asthma, which seemed to be a reliable predictive sign of the risk of occupational asthma [21,22].

The occupations most at risk appear to be those in the manufacture of resin-bound flexible foams [29,47] and the use of spray paint [48]. In Australia in 2014, the occupations with the highest proportion of employees exposed to isocyanates were still painters (34.7%), woodworkers (24.4%) and construction workers (17.7%) [27].

The data concerning the most harmful type of isocyanate and exposure are contradictory: MDI would be more asthmatogenic in the inhalation test because of its rapid polymerization [25,27], but it would be less dangerous than TDI because it is less volatile and not heated. Similarly, the use of oligomers and prepolymers would seem to be preferable to isocyanate monomers, the latter being more easily inhaled [27]. The risk of occupational asthma would seem to be highest for di-isocyanates (two groups on the same molecule), while the risk is not clearly established for mono-isocyanates [48]. In 2012, a literature review reported that long-term, low-intensity exposures did not appear to be more of a risk for occupational asthma as short, high exposures [49], whereas other studies reported that it is the duration of daily exposure that is predictive for the risk of occupational asthma [17,50]. However, it appears that exposures at undetectable levels may result in pulmonary symptoms such as tightness of the chest [23].

### 4.1. Limitations of the Review

The quality of the occupational asthma epidemiological studies was often insufficient and well-conducted prospective cohort studies often lacked power, limiting the precision of their results. In addition, they mostly had short follow-up periods, which are not compatible with the latency of occupational asthma, and therefore tended to underestimate its real incidence. Another limitation frequently stated by the authors of these cohort studies is the large number of dropouts during the follow-up period, which leads to the suspicion of a ‘healthy worker effect’ bias, due to patients who are most likely to have respiratory symptoms either not accepting to participate at recruitment or by dropping out during the study. One study reported that those lost to follow-up, because they left their job after a short period, were often in poorer health than long-term employees [10].

In comparison, the retrospective studies, with potentially very long follow-up, are intrinsically subject to information and recall bias and therefore, the accuracy of the conclusions drawn is less robust. Finally, the literature reviews analyzed synthesized highly variable and not easily comparable data, as the included studies were generally disparate.

However, all these limitations are inherent to occupational health studies and do not call into question the health effects observed. It should be emphasized that the comparison between studies is often difficult because of their non-uniform methodology. We have analyzed data from different types of studies, which were conducted in dissimilar settings, and which included populations that were not always representative of the populations actually exposed to isocyanates. For example, subjects with a pneumological history were excluded from some studies, even though they had been assigned to high-risk occupations, and in some studies, participants had previously been exposed to isocyanates [7,10].

The type of isocyanate studied, which varied between studies, is also a potential confounding factor. The diagnostic criteria used for asthma varied between studies, ranging from a simple declaration of symptoms by questionnaire, without medical examination to more formal tests such as PFT or specific bronchial provocation tests. Finally, as environmental measurements were not systematically reported, it is impossible to estimate with precision the degree of exposure in each study.

This heterogeneity observed between the various published studies is the consequence of the persistently poor understanding of the pathophysiology of the disease, a non-consensual diagnostic strategy, and non-uniform prevention measures used in different countries. The causal link with the occupational environment should also be more rigorously defined in a standardized manner in studies.

Moreover, we cannot exclude the possibility that the deliberately restrictive inclusion criteria, used to target a very precise subject, led to the non-inclusion of studies that may have reported data that would have been useful for this review. In addition, we limited the review to publications in French and English published in the last 30 years, which may have resulted in an incomplete literature review.

### 4.2. Conclusions

Occupational asthma caused by isocyanates is still a current occupational health problem, and its reduction over time has stagnated for several years despite the implementation of individual and collective preventive measures. Several animal studies included in the literature reviews that we analyzed have shown that sensitization to isocyanates in animals is possible by the cutaneous route [18,20]. In humans, we currently have only indirect evidence of pulmonary sensitization by this route, i.e., urinary biomarkers and HDI-conjugated keratin found in employees whose workplace was subject to analytical measurements of respiratory exposure below the detection threshold [32,51]. It would be very useful to study this exposure route more closely, as it could explain why the incidence of isocyanate-related occupational asthma seems to have plateaued. This could then help to define which skin protection equipment would be most effective against these agents.

We also need to develop precise, rigorous and reproducible methods that should be used in future cohort studies. This common methodology would enable us to perform meta-analyses with acceptable power to compare the relative risk for isocyanate used between different occupations and different types of industry.

## Figures and Tables

**Table 1:** Occupational asthma related to isocyanate exposure in the automotive industry.

Study ID (First Author, Year) [REF]	Study Design/Location (N)	Isocyanate	Follow-Up	Asthma Diagnosis	Causality Assessment	Results	Limitations
Simpson 1996 [7]	Case series/no details (34)	BIC	None	PFT + methacholine or bronchodilator challenge test	Temporality PFT	41% suspected OA cases after 10 weeks exposure without IPE All cases resolved after introduction of IPE	Exposure to BIC not measured Previous exposure for four subjects
Cullen 1996 [12]	Cross-sectional/New Haven, CT, USA (102)	HDI	None	Questionnaire Peak flow	Occupational exposure matrix	19.6% had respiratory symptoms Proportional to extent of exposure (35.7% vs. 6.7%) Atopy not associated with risk The use of air-supply masks was associated with risk reduction No link between questionnaire and change in PFT Increased risk in small, confined spaces Often sporadic forms of asthma	Insufficient IPE (surgical mask) Possible healthy worker bias Non-specific questionnaire (Modified ATS)
Ucgun ^a^ 1998 [8]	Observational/Eskisehir, Turkey (312)	TDI	None	Questionnaire PFT Peak flow Methacholine challenge test	Temporality	OA incidence for painters = 9.6% OA incidence proportional to the length of exposure.	Non-optimal use of IPE
Redlich 2002 [10]	Cohort/New Haven, USA (45)	HDI	One year (2001)	Questionnaire PFT Methacholine challenge test Serology	Declarative	PFT and stable symptoms after one-year exposure No OA cases during one-year follow-up	Small sample size Exposure not measured Short follow-up Previous exposure Unclear diagnosis
Hur 2008 [13]	Observational/South Korea (58)	MDI	None	Questionnaire Specialist consultation Prick tests ELISA PFT BCT	Temporality	22.4% of subjects symptomatic 8.6% OA Poor respiratory symptoms and presence of specific IgG were prognostic factors for the development of OA	
Stocks 2015 [9]	Cohort/UK (15,006)	HDI	8 years (2006–2014)	Notification of OA to SWORD register	Urine assay	Concomitant decreases in positive urine samples and isocyanate OA cases: prognostic factor?	Underreporting bias
Dey 2017 [11]	Cross-sectional/Assam, India (60)	ND	None	Questionnaire PFT	Work history	PFT reduction (OLD ± RLD) proportional to exposure duration	Exposure not measured Small sample size

^a^ Also included furniture painters. Abbreviations: ATS: American Thoracic Society; BIC: 1,3-bis(isocyanatomethyl)cyclohexane pre-polymer; BCT bronchial challenge test; HDI: hexamethylene diisocyanate; IPE: individual protection equipment; MDI: diphenylmethane 2,4-diisocyanate; ND: no details; OA: occupational asthma; OLD: obstructive lung disorder; PFT: pulmonary function test; RLD: restrictive lung disorder; TDI: 2,4 and 2,6 toluene diisocyanate.

**Table 2:** Isocyanate-related occupational asthma in the chemical industry.

Study ID (First Author, Year) [REF]	Study Design/Location (N)	Isocyanate	Follow-Up	Asthma Diagnosis	Causality Assessment	Results	Limitations
Kraw & Tarlo 1999 [14]	Case series/Ontario, Canada (39)	MDI/TDI	Five years (1992–1997)	Prick test Methacholine challenge test Peak flow	Temporality	One and two confirmed and suspected OA cases, respectively, for 39 subjects in five years	Small sample size Spontaneous consultations Sensitive but non-specific questionnaire
Ott et al., 2000 [15]	Retrospective cohort/Louisiana, USA (313)	TDI	Minimum five years (1967–1992)	Medical files Symptoms PFT	Medical files	6.4% suspected OA cases, from files Concentration and cumulative dose had an impact on PFT only in women More cases among new employees and those with a history of TDI exposure	Selection bias Reported symptoms Data from medical files
Cassidy et al., 2010 [16]	Retrospective cohort/southern USA (100)	HDI	Minimum two years (1987–2007)	Consultation PFT (FRV1)	Temporality Measures of exposure	FEV1 and FVC are lower in those unexposed aged >19 years Reduction in PFT results occurs more rapidly in unexposed subjects No OA cases No increase in skin sensitization	Possible healthy worker bias Smoker status not taken into account
Hathaway et al., 2014 [17]	Retrospective cohort/southern USA (73)	HDI	Up to 20 years (1991–2011)	Medical files Symptoms PFT Questions to the patient or last attending physician	Medical files Air monitoring records	No OA cases No exposure above exposure limit values Occasional and brief exposure accidents appear benign	No control group No biomonitoring Small sample size
Gui et al., 2014 [18]	Inception cohort/Eastern Europe (49)	TDI	One year (dates not given)	Questionnaire PFT	Serology Measures of exposure	14.2% subjects symptomatic after one-year exposure No exposure above exposure limit values No OA Potential skin sensitization	Prior exposure is not well defined Initial PFT not systematic Short study period Small sample size
Collins et al., 2017 [19]	Cohort/Texas and Louisiana, USA (197)	TDI	Five years (2007–2012)	Questionnaire PFT	Symptoms suggesting OA formal diagnosis	OA incidence = 0.009 cases/person-year OR = 2.08 (95% CI 1.07 to 4.05) per logarithm parts per billion-years and peak TDI exposures OR = 1.18 (95% CI 1.06 to 1.32)	Potential selection bias because fixed-term contract workers not included No systematic medical examination

Abbreviations: FEV1: forced expiratory volume; FVC: forced vital capacity; HDI: hexamethylene diisocyanate; IPE: individual protection equipment; MDI: diphenylmethane 2,4-diisocyanate; ND: no details; OA: occupational asthma; PFT: pulmonary function test; TDI: 2,4 and 2,6 toluene diisocyanate.

**Table 3:** Isocyanate-related occupational asthma in the wood industry.

Study ID (First Author, Year) [REF]	Study Design/Location (N)	Isocyanate	Follow-Up	Asthma Diagnosis	Causality Assessment	Results	Limitations
Petsonk et al., 2000 [20]	Inception cohort/ND (178)	MDI	Two years (1995–1996)	Questionnaire PFT Methacholine challenge test Peak flow Serology	Self-reported Exposure not measured	12% of exposed workers were symptomatic Symptomatology highly correlated with reported exposure, correct use of IPE, and reported skin exposure	Symptoms and exposure were self-reported and not very specific
Wang & Petsonk 2004 [21]	Inception cohort/ND (132)	MDI	Two years (1995–1996)	Symptoms reported in the questionnaire	Study of job type Exposure not measured	Main symptoms = wheezing, chest tightness, cough, dyspnea	Study based on the appearance of symptoms Confusion bias between different symptoms Poorly comparable groups Missing data No exposure measure

Abbreviations: IPE: individual protection equipment; MDI: diphenylmethane 2,4-diisocyanate; PFT: pulmonary function test.

**Table 4:** Isocyanate-related occupational asthma in foundries and molding plants.

Study ID (First Author, Year) [REF]	Study Design/Location (N)	Isocyanate	Follow-Up	Asthma Diagnosis	Causality Assessment	Results	Limitations
Bernstein et al., 1993 [22]	Cohort/Cincinnati, USA ^a^ (243)	MDI	Three years (1991–1993)	Questionnaire Immunological assays Peak flow Methacholine challenge test	Peak flow evaluation by a physician Temporality Continuous monitoring	Prevalence of OA = 1% for exposure < 0.05 ppb Prevalence decreased proportionally with exposure intensity	Peak flows too soon after avoidance Non-specific questionnaire
Cherry et al., 2002 [23]	Retrospective cohort/England ^b^ (991)	ND	Five years (dates not given)	Bronchial challenge test PFT Peak flow	Questionnaire Undetectable exposure measurements	No increased risk of PA if measurements remain undetectable Chest tightness reported by 16.1% of exposed individuals	Exposed women not included

^a^ Molding plant; ^b^ foundries. Abbreviations: MDI: diphenylmethane 2,4-diisocyanate; ND: no details; OA: occupational asthma; PFT: pulmonary function test.

**Table 5:** Isocyanate-related occupational asthma in other industries.

Study ID (First Author, Year) [REF]	Study Design/Location (N)	Isocyanate	Type of Industry	Follow-Up	Asthma Diagnosis	Causality Assessment	Results	Limitations
Park et al., 1992 [24]	Transversal study/South Korea (31)	TDI	Textiles industry	No follow-up	Questionnaire Prick tests RAST Methacholine challenge test	Specific inhalation test	26% of subjects were symptomatic 13% of inhalation tests were positive	Duration of exposure not provided
Baur et al., 1994 [25]	Retrospective cohort/Germany (1780)	MDI/TDI	Various	ND	Medical files Inhalation test	Specific inhalation test	MDI is more asthmatogenic because not easily measured and polymerizes rapidly 1–2% of exposed subjects were symptomatic	Archived data Diverse population
Al-Batanony et al., 2012 [26]	Transversal study/Egypt (100)	TDI	Bone-glue factory	No follow-up	Questionnaire Symptoms PFT	Urinary hippuric acid Absence of temporality	Impaired spirometry (FEV1 and FVC) proportional to the exposure time Exposure time correlated with the severity	Poorly comparable groups Link to work not clear
El-Zaemey et al., 2018 [27]	Descriptive study	MDI/TDI	Australian workers from all sectors aged 18–65 years	No follow-up	Evaluation of exposure prevalence	Self-reported via questionnaire	MDI less volatile than TDI. Most exposed trades: painting, wood, construction	Non-response bias Self-reported data No exposure measurement

FEV1: forced expiratory volume; FVC: forced vital capacity; MDI: diphenylmethane 2,4-diisocyanate; ND: no details; OA: occupational asthma; PFT: pulmonary function test; RAST: radioallergosorbent test; TDI: 2,4 and 2,6 toluene diisocyanate.

**Table 6:** Literature reviews in mixed populations assessing isocyanate-related occupational asthma.

Study ID (First Author, Year) [REF]	Isocyanate	Number of Studies Included	Follow-Up	Asthma Diagnosis	Causality Assessment	Results	Limitations
Van Kampen et al., 2000 [28]	All	300	No follow-up	PFT Inhalation test	Questionnaire	Prevalence respiratory disease = 5%	Possible selection bias Non-exhaustive review No assessment of study quality No comparison between substances and reported symptoms
Diller 2002 [29]	TDI	19	Variable	Diverse	Occupational history	Prevalence >10% before 1985, and mostly between 0 and 10% in recent years	Unclear selection criteria Populations and study dates varied Unclear diagnosis
Ott et al., 2003 [30]	TDI	ND	No follow-u	ND	ND	OA incidence decreased from 5% in 1970 to <1% No avoidance after symptom onset correlated with impaired lung function tests TDI-induced asthma causes bronchospasm even at 1 ppb	
Jarvis et al., 2005 [31]	All	ND	Variable	Clinical diagnosis	Diverse	Risk increased with the number of groups No mono-isocyanate-induced OA reported	Possible interactions between substances
Bello et al., 2007 [32]	All	800	No follow-up	Diverse	Reported cutaneous exposure	Indirect evidence of skin sensitization	Measurement of cutaneous exposure difficult Included animal studies

ND: no details; OA: occupational asthma; PFT: pulmonary function test; TDI: 2,4 and 2,6 toluene diisocyanate.

**Table 7:** Regional or national register studies assessing data related to occupational asthma due to any isocyanate and other causative agents.

Study ID (First Author, Year) [REF]	Location	Population	Participants	Follow-Up	Asthma Diagnosis	Causality Assessment	Results	Limitations
Gannon & Burge 1993 [33]	West Midlands, UK	Workers in the region	Cases reported by volunteer specialists	1989–1991	Peak flow	Actual or previous exposure at time of diagnosis	Incidence rate = 43/M active workers; 1883/M car body painters Isocyanates first cause (20.4%) Mainly allergic mechanism	Under-reporting bias Diagnostic methods underused outside specialized centers A region with high industrial activity
McDonald et al., 2000 [34]	UK	UK working population	Cases reported by volunteer specialists	1989–1997	Physician-dependent	Physician-dependent	Incidence rate = 1464/M car body painters Reduction in isocyanate-related OA since 1991 (from 22 to 14%)	Under-reporting bias
Esterhuizen et al., 2001 [35]	South Africa	All working population	Cases reported by volunteer specialists	1997–1999	Peak flow IgE Prick tests	Temporality	Incidence rate = 17.5/M active workers Main causative agents: isocyanates (16.7%) and flour (16.0%) The sector most at risk: health (16.4%)	Under-reporting bias Inconsistent diagnostic methods RADS classified as OA
Hnizdo et al., 2001 [36]	South Africa	All working population	Cases reported by volunteer specialists	1996–1998	Physician-dependent	Physician-dependent	Incidence rate = 13.1/M active workers Main causative agents: latex (24.1%) and isocyanates (19.5%)	Under-reporting bias Inconsistent diagnostic methods
Kopferschmitt-Kubler et al., 2002 [37]	France	All working population	Cases reported by volunteer specialists	1996	Peak flow IgE Prick tests Bronchial methacholine challenge test	Temporality	Incidence rate = 25.7/M active workers Main causative agents: flour (23.3%) and isocyanates (16.6%)	Under-reporting bias RADS classified as OA
Ameille et al., 2003 [38]	France	All working population	Cases reported by volunteer specialists	1996–1999	Physician-dependent	Physician-dependent	Main causative agents: flour (20.3%) and isocyanates (14.1%) Incidence rate = 24/M active workers Sectors most at risk: car body painter (326/M) and woodworkers (218/M)	Under-reporting bias Inconsistent diagnostic methods
Di Stefano et al., 2004 [39]	West Midlands, UK	All working population	Cases reported by volunteer specialists	1990–1997	Peak flow Bronchial methacholine challenge test	Temporality	Main causative agent: isocyanates (17.3%) Incidence rate = 41.2/M active workers The sector most at risk: car body painter Increasing avoidance after diagnosis (82.3% vs. 71.7%)	Under-reporting bias A region with high industrial activity
Vandenplas et al., 2005 [40]	Belgium	All working population	Cases reported by volunteer specialists	2000–2002	BHR IgE Prick tests PFT Bronchial methacholine challenge test	Temporality	Main causative agent: isocyanates (17.3%) Incidence rate = 23.5/M active workers	Under-reporting bias
Bakerly et al., 2008 [41]	West Midlands, UK	All working population in the region	Cases reported by volunteer specialists	1991–2005	Peak flow	Temporality Monitoring	Main causative agent: isocyanates (21%) Incidence rate = 42/M active workers No change in incidence in the study period. The sector most at risk: car body painter	Under-reporting bias
Mackie 2008 [42]	UK	Motor vehicle repair workers	Cases reported by volunteer specialists	1995–2000	Questionnaire PFT	Temporality	Incidence rate = 79/M motor vehicle repair workers 8/9 spray paints contain isocyanates No diagnostic benefit from PFT vs. questionnaire and temporality One in two cases still in work one year after diagnosis	Under-reporting bias High rate of lost-to-follow-up
Vandenplas et al., 2011 [43]	Belgium	Workers in Belgium	Retrospective review of compensation claims	1993–2002	Questionnaire PFT Prick tests Bronchial methacholine challenge test	Temporality	Main causative agents: flour (33.6%) et isocyanates (19.6%) Incidence rate = 29.4/M active workers The rate decreased from 35.5 to 25.8	Under-reporting bias Inconsistent diagnostic methods
Paris et al., 2012 [44]	France	All working population	Cases reported by specialists	2001–2009	Questionnaire Specialist consultation	Medical files	The incidence rate of isocyanate-related OA decreased from 64 (12.7%) à 15 (6.2%)	Under-reporting bias Inconsistent diagnostic methods
Reilly et al., 2020 [45]	Michigan, USA	All working population	Cases reported by volunteer specialists	1988–2018	Medical files Telephone interviews	Monitoring	Main causative solvents and isocyanates Incidence rate = 35/M active workers in 1988 20/M active workers in 2018 20% of workers exposed to isocyanates in 1988 vs. 7% in 2018 The sector most at risk: manufacturing	Under-reporting bias

BHR: bronchial hyperresponsiveness; ND: no details; OA: occupational asthma; PFT: pulmonary function test; RADS: reactive airways dysfunction syndrome.

## Data Availability

Not applicable.
